# The Basophil Activation Test in Allergy Diagnostics: Clinical Applications, Methodological Challenges, and Proposed Clinical Frameworks

**DOI:** 10.3390/cells15141241

**Published:** 2026-07-09

**Authors:** Marina Izmailovich, Aizhan Kabyldina, Zamira Seilkhan, Aruzhan Zhussip, Raushan Kozhanova, Tair Nurpeissov, Gulzada Uteubaeva, Olga Kazimirova, Alyona Lavrinenko, Lyubov Brizitskaya, Elina Suleimanova, Zarina Shaikhina

**Affiliations:** 1 Department of Internal Medicine, Karaganda Medical University, Karaganda 100000, Kazakhstan; 2 School of Dentistry, Karaganda Medical University, Karaganda 100000, Kazakhstan; 3 General Immunology, Asfendiyarov Kazakh National Medical University, Almaty 050000, Kazakhstan; 4 Internal Diseases No. 3, Astana Medical University, Astana 010000, Kazakhstan; 5 Department of Family Medicine, Karaganda Medical University, Karaganda 100000, Kazakhstan; 6 Scientific Research Laboratory, Institute of Life Sciences, Karaganda Medical University, Karaganda 100000, Kazakhstan; 7 Department of Oncology and Radiation Diagnostics, Karaganda Medical University, Karaganda 100000, Kazakhstan; 8 School of General Medicine, Karaganda Medical University, Karaganda 100000, Kazakhstan

**Keywords:** basophil activation test, BAT, allergy, IgE-mediated reactions, flow cytometry, food allergy, drug hypersensitivity, anaphylaxis, CD63, CD203c

## Abstract

The basophil activation test (BAT) is a modern in vitro functional assay used for the diagnosis of immunoglobulin E-mediated allergic reactions. The method is based on flow cytometric assessment of activation marker expression on peripheral blood basophils after stimulation with specific allergens. BAT has gained increasing clinical relevance in allergology, particularly in patients with anaphylaxis risk, polysensitization, drug hypersensitivity, and inconclusive skin or serological test results. This narrative review summarizes current evidence on the diagnostic performance and clinical applications of BAT in food allergy, drug hypersensitivity, Hymenoptera venom allergy, latex sensitization, and allergen immunotherapy monitoring. The review discusses the immunological mechanisms of basophil activation, the role of CD63 and CD203c, methodological aspects of the assay, sensitivity and specificity data, and advantages and limitations compared with conventional diagnostic approaches. Particular attention is given to protocol standardization, interpretation criteria, and the problem of non-responder patients. Current evidence indicates that BAT demonstrates high specificity and provides functional assessment of clinically relevant allergic reactions. In addition, this review proposes practical clinical frameworks for integrating BAT into allergy diagnostic pathways and for managing inconclusive or non-responder BAT results. Further standardization, multiplex formats, and automated analytical approaches may expand its role in personalized allergy diagnostics.

## 1. Introduction

Allergic diseases are among the most common immune-mediated disorders and continue to increase in prevalence worldwide. Food allergy, bronchial asthma, drug hypersensitivity, and anaphylaxis contribute substantially to healthcare burden because of their impact on quality of life, long-term disease management, and, in some cases, the risk of severe or life-threatening reactions [1,2]. As allergic diseases become increasingly heterogeneous in their clinical presentation, the need for diagnostic methods that are not only accurate but also clinically meaningful remains particularly important.

Skin prick testing and measurement of serum allergen-specific immunoglobulin E (IgE) continue to represent the foundation of allergy diagnostics. However, both approaches have well-recognized limitations. Skin testing may be difficult or unsafe in patients with severe allergic disease, concomitant skin disorders, or ongoing antihistamine therapy. Similarly, serological assays provide evidence of sensitization but do not necessarily determine whether sensitization is clinically relevant [3,4]. In clinical practice, discrepancies between laboratory findings and patient history are not uncommon, particularly in polysensitized individuals and in cases involving severe or equivocal reactions. In such situations, component-resolved diagnostics (CRD) may provide additional diagnostic precision by distinguishing true primary sensitization from cross-reactivity and by helping differentiate co-sensitization from clinically irrelevant sensitization patterns. The integration of molecular allergen components with conventional testing may therefore improve diagnostic accuracy, particularly in polysensitized patients and in cases where clinical history and routine laboratory findings appear discordant [5,6].

In this context, the basophil activation test (BAT) has become an area of growing clinical interest. BAT is an in vitro functional assay based on stimulation of circulating basophils with suspected allergens followed by flow cytometric assessment of activation markers, most commonly CD63 and CD203c. Unlike conventional methods that primarily identify sensitization, BAT evaluates the functional responsiveness of effector cells directly involved in allergic inflammation and may therefore provide additional information regarding the clinical significance of sensitization [3,7]. This may be especially relevant in diagnostically challenging situations, where conventional testing alone does not adequately explain the patient’s clinical presentation.

Over the last decade, BAT has been investigated in a broad range of allergic conditions, including food allergy, Hymenoptera venom allergy, drug hypersensitivity, latex sensitization, and severe allergic reactions associated with anaphylaxis risk. The method has also been investigated as an adjunctive tool for monitoring allergen immunotherapy and improving risk stratification in selected patient groups [7,8,9,10,11]. At the same time, several factors continue to limit broader implementation of BAT in routine clinical practice. Considerable variability remains in allergen preparations, stimulation protocols, basophil identification strategies, activation markers, cut-off values, and interpretation criteria, making reproducibility and inter-study comparison challenging [3,12].

This review summarizes current evidence regarding the immunological basis, methodological aspects, diagnostic performance, and clinical applications of BAT in allergy diagnostics. Particular attention is paid to methodological standardization, the interpretation of CD63 and CD203c expression, food allergy, drug hypersensitivity, allergen immunotherapy monitoring, and the non-responder phenomenon. Practical considerations related to implementation in resource-limited healthcare settings and the evolving role of BAT in precision allergology are also discussed. 

Beyond summarizing the available evidence, this review integrates current knowledge into a clinically oriented framework encompassing the diagnostic performance, methodological challenges, clinical implementation, and future directions of the basophil activation test. Particular emphasis is placed on the critical appraisal of the available evidence, the practical integration of BAT into multimodal allergy diagnostics, and the interpretation of inconclusive and non-responder results. The proposed evidence-informed clinical frameworks are intended to facilitate clinical decision-making while highlighting priorities for future research, validation, and methodological standardization.

## 2. Materials and Methods

A narrative review was conducted using a structured literature search and PRISMA-informed study selection process. The objective was to summarize current evidence regarding the immunological basis, methodological aspects, diagnostic performance, clinical applications, limitations, and future perspectives of the BAT in allergy diagnostics.

### 2.1. Search Strategy and Data Sources

A comprehensive literature search was conducted in PubMed/MEDLINE, Scopus, and Google Scholar databases covering the period from January 2016 to March 2026. The search strategy combined Medical Subject Headings (MeSH) and free-text keywords related to the basophil activation test and allergic diseases. The following search terms and Boolean operators were used: “basophil activation test”, “BAT”, “allergy diagnostics”, “food allergy”, “drug hypersensitivity”, “anaphylaxis”, “Hymenoptera venom allergy”, “latex allergy”, “allergen immunotherapy”.

The search strategy was adapted according to the requirements of each database. Additional relevant publications were identified through manual screening of reference lists from key reviews, systematic reviews, position papers, and international guidelines. Only articles published in English were considered eligible for inclusion. The search was limited to human studies and publications available in full text.

Because of considerable variation in allergen preparations, stimulation protocols, basophil identification strategies, activation markers, study populations, and outcome measures, direct comparison across studies remains challenging. Therefore, the available literature was synthesized in a clinically oriented narrative format, with emphasis on methodological considerations, diagnostic interpretation, and practical applicability in allergy care.

### 2.2. Eligibility Criteria

Studies were considered eligible for inclusion if they were published in English, involved human subjects, and addressed the clinical application, methodology, activation markers, or diagnostic performance of BAT in allergic diseases. Eligible publication types included original clinical studies, systematic reviews, meta-analyses, expert consensus documents, position papers, and international guidelines. Studies reporting sensitivity, specificity, predictive values, clinical utility, or methodological aspects relevant to BAT interpretation were prioritized.

Publications were excluded if they were non-English articles, animal studies, conference abstracts, editorials, comments, letters, case reports, studies with insufficient BAT-related clinical data, methodological papers without direct clinical applicability, or publications without accessible full text.

### 2.3. Study Selection and Data Synthesis

A total of 2240 records were identified through database searching, including PubMed (*n* = 628), Scopus (*n* = 1253), and Google Scholar (*n* = 359). After removal of 872 duplicate records, 1368 publications remained for title and abstract screening. During the screening phase, 728 records were excluded because of irrelevant topics, animal studies, conference abstracts, comments, letters, case reports, or unavailable full texts.

**Figure 1:**
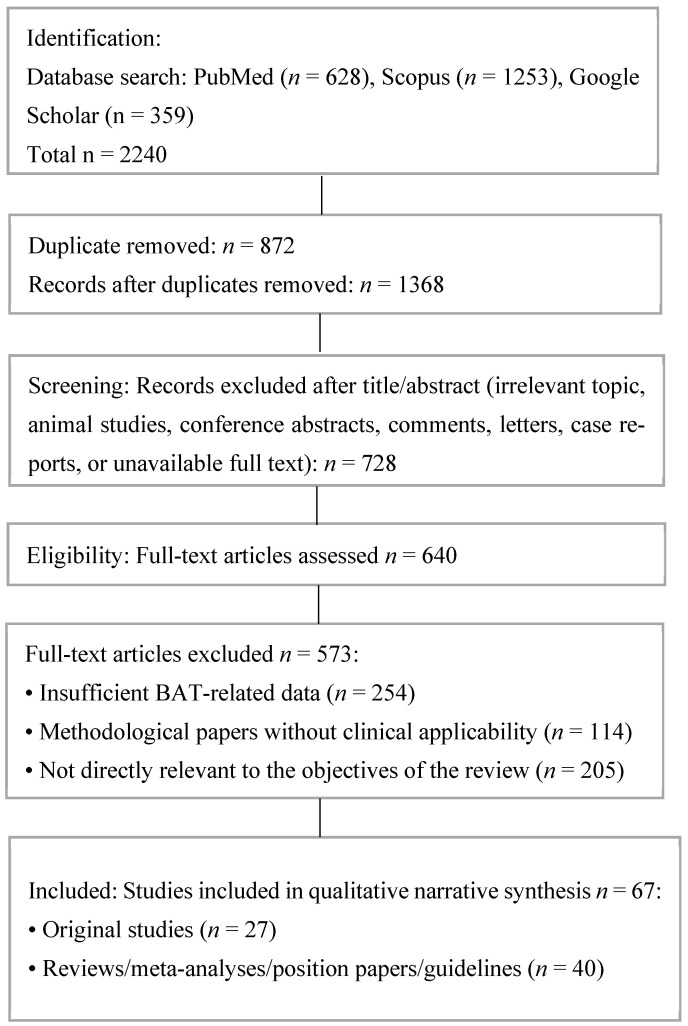
PRISMA flow diagram of the study selection process.

A total of 640 full-text articles were assessed for eligibility. Of these, 573 publications were excluded due to insufficient BAT-related data (*n* = 254), methodological focus without direct clinical applicability (*n* = 114), or lack of relevance to the objectives of the review (*n* = 205). Ultimately, 67 studies met the eligibility criteria and were included in the final qualitative synthesis. The study selection process is summarized in Figure 1.

### 2.4. Data Synthesis and Development of Clinical Frameworks

The included studies were synthesized using a qualitative narrative approach. Evidence from original clinical studies, systematic reviews, meta-analyses, expert consensus documents, and international guidelines was integrated to establish a comprehensive conceptual framework describing the current role of the BAT in allergy diagnostics. Table 1 summarizes selected representative original clinical studies that illustrate the diagnostic performance and principal clinical applications of BAT across different allergic diseases. The remaining eligible studies were incorporated into the narrative synthesis according to their thematic relevance. Findings related to the immunological basis of basophil activation, methodological aspects of BAT performance, diagnostic accuracy, clinical applications, and limitations were systematically compared and mapped across different allergic diseases and clinical scenarios. Particular emphasis was placed on identifying areas of established clinical utility, sources of methodological variability, challenges in result interpretation, and emerging directions for the implementation of BAT in precision allergology.

This qualitative synthesis enabled the integration of mechanistic, methodological, and clinical evidence into a unified evidence-based framework describing the position of BAT within contemporary diagnostic pathways for IgE-mediated allergic diseases. The conceptual models presented in this review, including the proposed approaches to BAT interpretation and management of non-responder or inconclusive results, were derived from the collective body of evidence identified during the literature review. These models are intended to facilitate clinical interpretation and highlight priorities for future research. They should not be considered validated clinical guidelines, but rather evidence-informed conceptual frameworks.

The studies summarized in Table 1 demonstrate generally favorable diagnostic performance of BAT across a range of allergic diseases. However, considerable heterogeneity exists with respect to study design, patient populations, allergen preparations, activation markers, and positivity thresholds, highlighting the need for careful interpretation of diagnostic accuracy estimates and further methodological standardization.

### 2.5. Critical Appraisal of the Included Studies

As shown in Table 1, the studies varied substantially in design, patient populations, and sample size. Although several investigations included relatively large cohorts and demonstrated consistent diagnostic performance of BAT, many studies were conducted in specialized tertiary allergy centers and involved highly selected patient populations. Consequently, selection bias and referral bias cannot be excluded.

Additional heterogeneity arose from differences in allergen preparations, stimulation protocols, activation markers, and positivity thresholds. These methodological differences may influence reproducibility and contribute to variation in reported sensitivity across studies. While BAT generally demonstrated high specificity irrespective of the clinical setting, variability in sensitivity estimates remains one of the most frequently reported findings in the literature.

Furthermore, the predominance of studies originating from specialized allergy centers may limit the generalizability of the findings to broader clinical practice. Continued efforts toward methodological standardization and multicenter validation are therefore required to support the wider implementation of BAT.

Because of the narrative nature of this review and the heterogeneity of the included study designs, no formal risk-of-bias assessment was performed. Instead, methodological quality, potential sources of bias, reproducibility, and generalizability were critically evaluated using a narrative approach.

## 3. Immunological Basis of Basophil Activation

### 3.1. Mechanisms of Basophil Activation in Allergy

Basophils play an important role in immediate hypersensitivity reactions and represent one of the key effector cell populations involved in IgE-mediated allergy. Although they account for less than 1% of circulating leukocytes, their capacity to rapidly release proinflammatory mediators makes them clinically relevant in the development of allergic symptoms [28,29,30]. 

During the sensitization phase, exposure to an allergen induces a T helper 2 (Th2)-dominant immune response characterized by the production of interleukin (IL)-4 and IL-13, which promote class switching and synthesis of allergen-specific IgE antibodies. These antibodies subsequently bind to the high-affinity Fc epsilon receptor I (FcεRI) expressed on the surface of basophils, thereby sensitizing the cells to future allergen exposure [28,29].

Upon repeated exposure to the same allergen, cross-linking of receptor-bound IgE initiates intracellular signaling pathways involving tyrosine kinases such as Lyn and Syk, followed by calcium influx and activation of downstream signaling cascades. This process ultimately leads to basophil activation and degranulation, resulting in the release of histamine, leukotrienes, prostaglandins, and cytokines that contribute to the development of allergic inflammation and clinical manifestations, including urticaria, angioedema, bronchospasm, and anaphylaxis [30,31,32,33]. 

Although IgE-mediated activation remains the best characterized pathway, basophils may also be activated through IgE-independent mechanisms, including complement activation and direct stimulation by selected drugs or inflammatory mediators. Recognition of these alternative pathways is clinically important, particularly in drug hypersensitivity, where basophil activation does not always reflect classical allergen-specific IgE responses [25,28,29,30,34,35,36].

From a diagnostic perspective, the biological rationale for BAT is based on the ability to reproduce allergen-induced basophil activation under controlled in vitro conditions. By measuring changes in activation-associated surface markers, particularly CD63 and CD203c, BAT provides insight into the functional responsiveness of effector cells and may therefore offer clinically relevant information beyond sensitization alone [3,7,8].

### 3.2. Basophil Activation in Food Allergy

Food allergy represents one of the clinical settings in which BAT has shown particularly strong diagnostic utility. In most cases, food allergy is mediated by immediate IgE-dependent hypersensitivity mechanisms, whereby exposure to a food allergen induces allergen-specific IgE production and sensitization of effector cells, including basophils and mast cells. Upon re-exposure, allergen-mediated cross-linking of receptor-bound IgE triggers cellular activation and release of inflammatory mediators responsible for symptoms ranging from mild urticaria and gastrointestinal manifestations to severe systemic reactions, including anaphylaxis [32,36,37]. 

Within this context, BAT provides a functional assessment of basophil responsiveness to specific food allergens under controlled in vitro conditions. This feature is particularly valuable because conventional diagnostic approaches, including skin prick testing and allergen-specific IgE measurement, do not always distinguish clinically relevant allergy from asymptomatic sensitization [3,4,5,7,8,37]. Such diagnostic uncertainty is especially common in polysensitized individuals, patients with low allergen-specific IgE concentrations, and cases in which clinical history and conventional test results are discordant.

In selected patients, BAT may be used alongside component-resolved diagnostics to improve characterization of allergen-specific sensitization profiles. While CRD provides molecular-level information regarding individual allergenic components and cross-reactive proteins, BAT offers complementary functional assessment of effector-cell activation. The combined use of these approaches may enhance diagnostic confidence and reduce uncertainty in complex food allergy cases [5,6,38].

Current evidence indicates that BAT may be especially useful when conventional diagnostic findings are inconclusive or when oral food challenge is associated with an increased risk of severe reactions. By evaluating effector-cell reactivity rather than sensitization alone, BAT may help improve diagnostic confidence and support more individualized clinical decision-making in selected patients with suspected food allergy. Diagnostic utility has been demonstrated across a broad spectrum of food allergens, including peanut, tree nuts, cow’s milk, hen’s egg, wheat, shrimp, and lipid transfer protein (LTP)-associated food allergy [14,15,16,23,37,39,40,41]. 

At the same time, BAT should not be interpreted as a universal replacement for established diagnostic methods. Its diagnostic performance may vary depending on the food allergen, patient age, allergen preparation, stimulation concentration, activation marker, and positivity threshold used. Therefore, broader integration of BAT into food allergy diagnostics requires further methodological standardization and validation across different clinical settings [5,6,13,37,38,42]. 

Although BAT has demonstrated consistently high diagnostic performance for peanut, tree nut, cow’s milk, and hen’s egg allergy, its clinical utility appears less well-established for several other food allergens because of the limited number of validation studies and smaller patient cohorts [6,13,14,15,16,20,37,38,39,40,41,42]. Furthermore, the diagnostic thresholds reported for individual food allergens are not directly interchangeable, indicating that allergen-specific interpretation remains essential [6,12,37,38,40,41,42]. These observations suggest that BAT should currently be integrated into food allergy diagnostics in a targeted, indication-specific manner rather than being applied uniformly across all suspected food allergies [5,6,37,38].

### 3.3. Basophil Reactivity During Allergen-Specific Immunotherapy

Allergen-specific immunotherapy (AIT) induces a range of immunological changes that gradually contribute to the development of immune tolerance. During the early stages of treatment, transient allergen-induced basophil activation may still occur; however, prolonged exposure to controlled allergen doses is generally associated with reduced cellular responsiveness. This process is thought to involve several immunological mechanisms, including increased production of allergen-specific IgG4 antibodies with blocking activity, reduced FcεRI expression on effector cells, modulation of allergen-specific IgE responses, and induction of regulatory immune pathways mediated by IL-10 and transforming growth factor beta (TGF-β) [7,8,11,43]. 

Because BAT directly evaluates allergen-induced basophil responsiveness, the method has attracted interest as a potential tool for monitoring immunological changes during AIT. A reduction in basophil activation over time has been associated with clinical improvement in several studies, suggesting that BAT may reflect the gradual establishment of immune tolerance [6,7,11]. At the same time, the relationship between changes in BAT results and long-term clinical outcomes remains incompletely understood, and findings across studies have not been entirely consistent.

From a clinical perspective, BAT may be especially useful as a complementary biomarker in selected situations, especially when objective assessment of treatment response is challenging. However, current evidence remains insufficient to support its routine use as a stand-alone tool for monitoring AIT efficacy. Variability in stimulation protocols, activation markers, treatment duration, and outcome measures continues to limit comparability across studies and complicates interpretation of longitudinal changes [11,12,44]. 

At present, BAT should be regarded primarily as an adjunctive method that may provide additional information regarding immunological response during AIT rather than a validated surrogate marker of sustained clinical benefit. Further prospective studies and greater methodological standardization will be necessary to better define its role in treatment monitoring and individualized therapeutic decision-making [11,12,44].

While BAT has demonstrated strong utility in food allergy, its application has expanded to other areas of allergology, particularly drug hypersensitivity. 

### 3.4. Basophil Activation in Drug Allergy

Drug hypersensitivity reactions represent a heterogeneous group of adverse immune-mediated responses, among which immediate IgE-mediated reactions are of particular clinical importance because of their potential severity. In this setting, small-molecule drugs or their metabolites may act as haptens by binding to endogenous proteins and forming antigenic complexes capable of inducing allergen-specific immune responses [34,35]. Under the influence of Th2-associated cytokines, drug-specific IgE antibodies may subsequently be produced and bind to FcεRI receptors expressed on the surface of basophils, thereby sensitizing effector cells to future drug exposure [28,29,34,35].

Upon re-exposure to the culprit drug, cross-linking of receptor-bound IgE initiates intracellular signaling pathways involving tyrosine kinases such as Lyn and Syk, followed by calcium mobilization and basophil activation. This process results in the release of inflammatory mediators that may contribute to clinical manifestations ranging from urticaria and angioedema to bronchospasm and anaphylaxis [30,31,34,35]. However, not all drug-induced reactions are mediated through classical IgE-dependent mechanisms. Certain drugs may activate basophils through alternative pathways, including complement-mediated activation or direct stimulation independent of allergen-specific IgE [34,35,45]. 

In addition to classical IgE-mediated mechanisms, certain drugs may induce immediate hypersensitivity-like reactions through activation of the Mas-related G-protein-coupled receptor X2 (MRGPRX2). This pathway has been implicated particularly in reactions associated with fluoroquinolones, selected neuromuscular blocking agents, and opioid compounds such as morphine. Unlike FcεRI-mediated activation, MRGPRX2-dependent responses may occur in the absence of prior sensitization and therefore represent a distinct mechanism of pseudoallergic or non-IgE-mediated hypersensitivity. Although BAT has demonstrated clinical utility in IgE-mediated drug allergy, its diagnostic value in reactions involving MRGPRX2 activation remains insufficiently established, and currently available evidence is limited [34,35].

**Figure 2:**
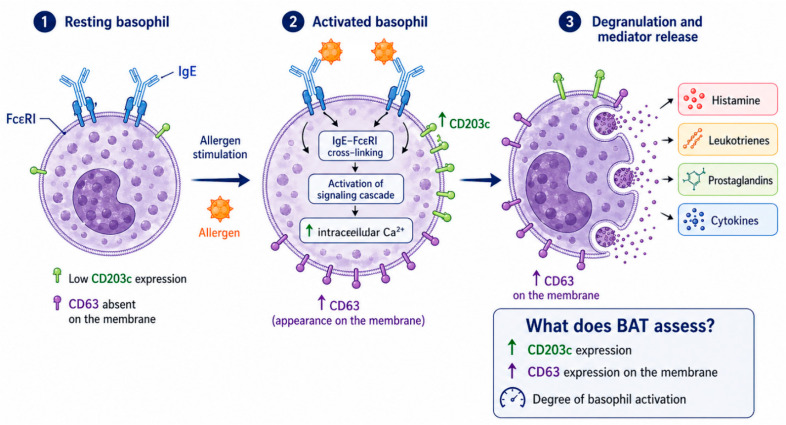
Schematic representation of allergen-induced basophil activation and BAT-associated marker expression, including increased CD203c expression, surface translocation of CD63, and release of inflammatory mediators. Dark blue arrows indicate progression between the stages of basophil activation, whereas black arrows indicate intracellular signaling and mediator release. Orange symbols represent allergens, green and purple membrane symbols indicate CD203c and CD63, respectively, and purple dots represent released granule contents and inflammatory mediators. Created by the authors with AI-assisted graphical support using ChatGPT (GPT-5.5 Thinking; OpenAI, San Francisco, CA, USA) and manually revised based on the published evidence [30,31,34,35].

A simplified overview of basophil activation and BAT-related marker expression is presented in Figure 2.

From a practical standpoint, BAT may be particularly useful in patients with suspected immediate drug hypersensitivity when conventional diagnostic findings are inconclusive or when drug provocation testing is associated with increased risk. This is especially relevant in patients with a history of severe systemic reactions, including drug-induced anaphylaxis, where in vivo testing may be unsafe [25,45].

At the same time, the diagnostic performance of BAT in drug allergy remains heterogeneous and appears to depend on several factors, including the drug class, immunological mechanism involved, timing of testing after the reaction, and the capacity of the drug or its metabolites to induce basophil activation in vitro. Consequently, BAT should currently be regarded as a complementary diagnostic method that may support clinical decision-making rather than a stand-alone alternative to established diagnostic approaches. Interpretation of results should always consider clinical history and, where appropriate, findings from skin testing and controlled drug provocation procedures [17,25,45].

It should be noted that the diagnostic utility of BAT varies substantially across different types of drug hypersensitivity reactions. While BAT may provide useful information in selected IgE-mediated reactions, particularly for some antibiotics and perioperative drugs, current evidence suggests limited clinical value in non-allergic hypersensitivity reactions to nonsteroidal anti-inflammatory drugs (NSAIDs). Because most NSAID hypersensitivity reactions are not mediated by allergen-specific IgE but rather by pharmacological mechanisms involving cyclooxygenase-1 inhibition and dysregulated eicosanoid metabolism, BAT generally demonstrates poor and inconsistent diagnostic performance in this setting. Therefore, BAT is not currently recommended as a routine diagnostic tool for NSAID hypersensitivity [34,45].

Similarly, the role of BAT in beta-lactam hypersensitivity should be interpreted within the context of established diagnostic pathways. In many patients with suspected immediate beta-lactam allergy, diagnosis can be reliably established through a combination of clinical history, skin testing, serum-specific IgE assessment, and, when appropriate, drug provocation testing. Therefore, BAT is generally regarded as a complementary rather than a primary diagnostic tool in beta-lactam allergy and may be most useful in selected cases with inconclusive conventional findings or when additional diagnostic confirmation is required [17,18,45].

The interpretation of BAT results in drug hypersensitivity remains particularly challenging because diagnostic performance differs substantially among individual drug classes and underlying immunological mechanisms. While BAT demonstrates high specificity in selected IgE-mediated reactions, its sensitivity is considerably more variable and remains insufficient for several non-IgE-mediated hypersensitivity phenotypes [17,18,25,34,45]. Consequently, the available evidence supports a drug-specific rather than a universal approach to BAT application, emphasizing the need for further validation across individual drugs and standardized testing protocols before broader implementation in routine drug allergy diagnostics [12,45].

## 4. Methodological Principles of BAT

The basophil activation test is most commonly performed using flow cytometry and is based on in vitro stimulation of circulating basophils with suspected allergens, followed by assessment of activation-associated surface marker expression. Although the overall analytical principle remains relatively consistent, considerable methodological variability exists across laboratories and published studies, affecting reproducibility and comparability of results [3,12,46,47].

A critical step in BAT is the accurate identification of basophils within peripheral blood samples. Because basophils represent a small fraction of circulating leukocytes, reliable gating strategies are essential for analytical accuracy. In most protocols, basophils are identified using combinations of markers such as CCR3, CD123, CRTH2, and FcεRI, together with exclusion of HLA-DR-positive cells. However, no universally accepted gating strategy currently exists, and differences in basophil identification continue to represent a source of inter-laboratory variability [12,46,47]. 

**Figure 3:**
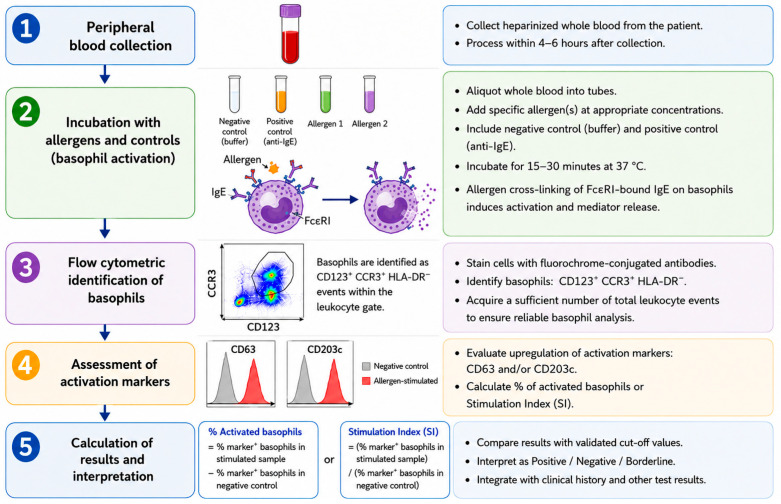
General workflow of the BAT, including peripheral blood collection, allergen stimulation, flow cytometric basophil identification, assessment of activation markers (CD63/CD203c), and interpretation of test results. Created by the authors with AI-assisted graphical support using ChatGPT and manually revised based on the published evidence [3,12,46,47].

Following basophil identification, cells are incubated with suspected allergens together with negative and positive controls. Stimulation induces changes in activation-associated surface markers, most commonly CD63 and CD203c, which are subsequently quantified by flow cytometry. CD63 is generally regarded as a marker of basophil degranulation and reflects granule–membrane fusion, whereas CD203c is considered an earlier activation marker that may increase even in the absence of complete degranulation [3,8,17]. Because these markers capture partly distinct aspects of cellular activation, their combined assessment may improve diagnostic sensitivity and provide a more comprehensive characterization of basophil responsiveness. A simplified workflow of the BAT procedure is presented in Figure 3.

Available evidence remains inconsistent regarding whether combined assessment of CD63 and CD203c provides clinically meaningful advantages over the use of a single activation marker, with reported performance varying according to the allergen, patient population, and laboratory methodology. Similarly, variability in positivity thresholds and laboratory protocols continues to complicate inter-laboratory standardization and comparison of published results, underscoring the need for further methodological harmonization before broader clinical implementation [12,17,46,47].

Despite increasing clinical interest in BAT, methodological heterogeneity remains one of the major barriers to broader implementation. Differences in allergen concentrations, incubation times, stimulation conditions, antibody panels, gating strategies, and cut-off values used to define positivity may substantially influence test performance. In addition, pre-analytical variables, including sample handling, storage conditions, and time from blood collection to analysis, may further affect basophil viability and responsiveness, potentially contributing to inconsistent or non-interpretable results [12,46,47,48]. Because basophil reactivity progressively declines after blood collection, BAT generally requires analysis of fresh samples within approximately 4 h to maintain optimal cellular responsiveness and analytical reliability.

At present, international efforts aimed at methodological harmonization and standardization of BAT protocols are ongoing. Greater consistency in analytical procedures, reporting standards, and clinically validated positivity thresholds will be essential not only to improve inter-laboratory reproducibility but also to facilitate comparison between studies and support broader implementation of BAT in routine allergy diagnostics [11,12,46,47].

## 5. Clinical Applications of BAT

The clinical use of BAT has expanded markedly, particularly in situations where conventional allergy diagnostics provide inconclusive or clinically discordant results. Because BAT evaluates allergen-induced functional responsiveness of basophils rather than sensitization alone, the method may provide additional diagnostic value in selected patient populations, especially when skin testing and allergen-specific IgE measurements are insufficient to establish diagnostic certainty.

Overall, current evidence indicates that BAT provides the greatest clinical benefit in food allergy, particularly when conventional diagnostic tests are inconclusive or discordant. The strongest evidence currently supports its use in peanut, tree nut, cow’s milk, and hen’s egg allergy, whereas evidence for other food allergens remains comparatively limited. By providing a functional assessment of allergen-induced basophil activation, BAT may reduce the need for oral food challenges in carefully selected patients. However, test results should always be interpreted in conjunction with clinical history, conventional allergy testing, and, when appropriate, component-resolved diagnostics [6,13,14,15,20,22,38].

In Hymenoptera venom allergy, BAT has shown diagnostic performance comparable to, and in some cases exceeding, that of skin testing and serological assays. In addition, the use of standardized antibody panels has been associated with improved reproducibility of BAT results across laboratories [49,50].

The diagnostic performance of BAT in drug hypersensitivity appears more heterogeneous and largely depends on the specific drug class and underlying immunological mechanism. In patients with suspected immediate hypersensitivity to beta-lactam antibiotics, BAT has demonstrated high specificity, supporting its potential role in confirming clinically relevant allergy and facilitating the delabeling of false drug allergy diagnoses [17,18,45]. However, the interpretation of BAT in drug hypersensitivity requires careful consideration of reaction phenotype, timing of testing, and the capacity of individual drugs or metabolites to induce basophil activation under in vitro conditions. Key studies supporting the diagnostic performance and clinical applications of BAT across different allergic diseases are summarized in Table 1.

BAT has also been investigated longitudinally, particularly in relation to allergen immunotherapy and risk stratification. Reduced basophil reactivity during treatment has been associated with clinical improvement and the development of immunological tolerance in some patient populations [6,11,26]. In addition, BAT is increasingly being explored as a potential tool for assessing reaction severity and anaphylaxis risk, particularly in severe food allergy [6,7]. Nevertheless, evidence regarding its predictive performance remains heterogeneous, and further validation in larger prospective studies is needed.

Taken together, the clinical value of BAT appears to be greatest in carefully selected clinical scenarios where conventional diagnostic methods do not adequately reflect the functional relevance of sensitization. This is particularly relevant in food allergy, selected forms of drug hypersensitivity, polysensitized patients, and situations in which provocation testing is contraindicated or associated with an increased risk of severe reactions. Accordingly, BAT should be regarded as a complementary second-line functional assay integrated into a multimodal diagnostic strategy rather than as a replacement for established diagnostic approaches. Its broader implementation in routine clinical practice will depend on continued methodological standardization, validation across diverse patient populations, and improved accessibility of testing platforms.

Importantly, the strength of evidence supporting BAT is not uniform across all allergic diseases. While its diagnostic utility has been consistently demonstrated in food allergy and Hymenoptera venom allergy, evidence remains comparatively limited for latex allergy, several drug classes, and some emerging clinical applications. Furthermore, differences in study design, patient selection, and reference standards continue to complicate direct comparison of published results. These considerations support the use of BAT as a targeted diagnostic tool in clinical settings where its performance has been adequately validated, while emphasizing the need for further high-quality studies in less well-established indications [3,5,8,12,17,18,37,45,49,50].

However, the clinical utility of BAT cannot be considered independently of its diagnostic performance, which varies according to the allergen, patient population, and methodological approach used across studies.

## 6. Diagnostic Accuracy and Limitations of BAT

### 6.1. Sensitivity and Specificity Across Allergic Diseases

The available evidence indicates that BAT demonstrates substantial diagnostic utility across several IgE-mediated allergic diseases; however, reported sensitivity and specificity vary considerably depending on the allergen, disease phenotype, patient population, and methodological approach used. Differences in allergen preparation, stimulation protocols, activation markers, gating strategies, and positivity thresholds continue to complicate direct comparison between studies and may partly explain variability in reported diagnostic performance [3,12,37,38,45,47]. 

Among allergic conditions, BAT appears to demonstrate the strongest diagnostic performance in food allergy, particularly peanut, tree nut, cow’s milk, and hen’s egg allergy, where high specificity and clinically meaningful sensitivity have been reported [6,13,14,15,38,42]. Similarly, in Hymenoptera venom allergy, BAT has shown diagnostic accuracy comparable to, and in some studies exceeding, that of conventional skin testing and serological assays [49,50].

In contrast, diagnostic performance in drug hypersensitivity remains more heterogeneous and appears to depend strongly on the drug class, immunological mechanism, and timing of testing following the reaction. Despite this variability, BAT generally demonstrates high specificity in selected clinical settings, supporting its usefulness in confirming clinically relevant hypersensitivity when conventional findings are inconclusive [17,18,25,45].

Although the reported diagnostic performance of BAT is encouraging, interpretation of sensitivity and specificity estimates should be approached cautiously. Considerable methodological heterogeneity across studies, together with differences in study design and patient selection, limits direct comparability and may reduce the generalizability of the findings to routine clinical practice [8,12,45,46,47].

### 6.2. Comparison with Conventional Diagnostic Methods

Conventional allergy diagnostics are primarily based on skin prick testing, measurement of serum allergen-specific IgE, and, when necessary, controlled provocation procedures. Although these methods remain the cornerstone of diagnostic evaluation, each has recognized limitations that may complicate interpretation in selected clinical settings [3,4,5,12]. A comparative overview of BAT and conventional allergy diagnostic methods is presented in Table 2.

In patients with LTP allergy, BAT has demonstrated considerable diagnostic value, with reported sensitivity exceeding 87% and specificity approaching 85%. Studies combining BAT with serological markers have also suggested improved diagnostic accuracy and more reliable stratification of clinically relevant allergy in selected patient groups. However, the ability of BAT to predict reaction severity in LTP allergy remains incompletely understood and continues to be investigated [23,40,41].

**Figure 4:**
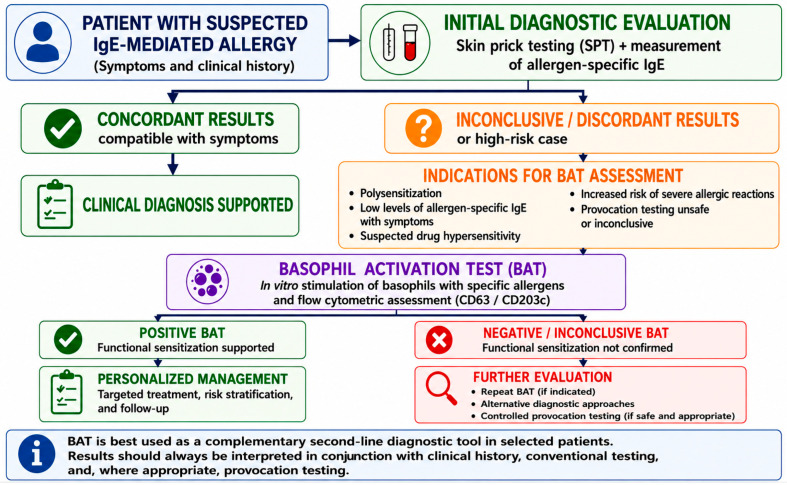
The framework represents an evidence-informed conceptual model and should not be interpreted as a validated clinical guideline. BAT may provide additional diagnostic value in patients with inconclusive or discordant conventional findings, polysensitization, suspected drug hypersensitivity, increased risk of severe allergic reactions, or situations in which provocation testing is unsafe or inconclusive. BAT should be interpreted as a complementary second-line diagnostic tool within a multimodal diagnostic approach. Created by the authors with AI-assisted graphical support using ChatGPT and manually revised based on the published evidence [3,5,6,12,18,45].

From a clinical standpoint, BAT appears to provide the greatest additional diagnostic value when conventional methods fail to adequately reflect the functional relevance of sensitization. This is especially important in polysensitized patients, individuals with low allergen-specific IgE levels, or cases involving discordant skin test and serological findings. Unlike conventional assays that primarily detect sensitization, BAT evaluates the functional responsiveness of effector cells and may therefore more closely reflect clinically relevant allergy. Nevertheless, BAT should be interpreted as part of a multimodal diagnostic approach rather than an isolated test, with results considered in conjunction with clinical history, conventional allergy testing, and, where appropriate, controlled provocation procedures [3,5,6,12]. A proposed clinical framework for integrating BAT into the diagnostic pathway of IgE-mediated allergic diseases is presented in Figure 4.

### 6.3. Advantages and Clinical Limitations of BAT

From a practical perspective, the principal advantage of BAT lies in its ability to provide functional evidence of clinically relevant allergen reactivity, thereby complementing conventional diagnostic methods that primarily detect sensitization. Consequently, BAT appears to be most valuable in carefully selected patients with diagnostically inconclusive or discordant findings, particularly when oral provocation testing is unsafe or associated with increased risk [3,5,6,12].

Furthermore, BAT appears to be less informative in immediate drug hypersensitivity reactions mediated through non-IgE pathways, including MRGPRX2-dependent mechanisms. In such cases, basophil activation may not accurately reflect the underlying biological processes, and the diagnostic performance of BAT remains insufficiently established. Consequently, negative BAT results should be interpreted cautiously when non-IgE-mediated drug hypersensitivity is suspected [34,35].

Despite its considerable clinical potential, several important limitations continue to restrict broader implementation of BAT in routine practice. The method requires fresh peripheral blood samples, strict pre-analytical handling, specialized flow cytometry equipment, and trained laboratory personnel. In most protocols, blood samples should ideally be processed within approximately 4 h of collection to preserve basophil viability and functional responsiveness. In addition, substantial methodological variability remains across laboratories, including differences in allergen preparations, stimulation protocols, gating strategies, activation markers, and positivity thresholds, all of which may influence diagnostic reproducibility and interpretation [12,46,47,48].

Another important limitation is the presence of so-called “non-responder” patients, whose basophils fail to respond adequately even to positive controls. This phenomenon may reduce the interpretability of BAT in a subset of patients and highlights the importance of appropriate quality control measures and cautious result interpretation [36,51]. 

Despite these limitations, BAT should be regarded as a complementary second-line functional assay within an integrated multimodal diagnostic strategy. Its principal value lies in resolving diagnostic uncertainty by providing functional evidence of clinically relevant sensitization in selected patients for whom conventional diagnostic approaches remain inconclusive [3,5,6,12,45].

### 6.4. Standardization Challenges

Despite increasing clinical interest and encouraging diagnostic performance across several allergic diseases, methodological standardization remains one of the principal barriers to the broader implementation of BAT. Considerable variability persists among laboratories with respect to allergen preparations, stimulation protocols, basophil identification strategies, activation markers, and positivity thresholds, limiting inter-study comparability and reproducibility of diagnostic results [3,12,46,47]. 

In addition to analytical variability, pre-analytical factors such as sample handling, transport conditions, and delays between blood collection and analysis may substantially influence basophil viability and responsiveness, emphasizing the importance of standardized laboratory procedures [46,47,48].

Current international initiatives, including EAACI position papers, standardized laboratory protocols, and external quality assurance programs, represent important steps toward harmonization of BAT methodology. Nevertheless, further multicenter validation studies and internationally accepted analytical standards will be required before BAT can be more broadly incorporated into routine clinical practice and evidence-based diagnostic algorithms [12,46,47,52].

### 6.5. Non-Responder Phenomenon

An important methodological and clinical limitation of BAT is the presence of so-called “non-responder” patients, whose basophils fail to respond adequately even to positive controls, thereby limiting the interpretability of BAT results and contributing to diagnostic uncertainty [36]. The mechanisms underlying non-responsiveness are likely multifactorial and may involve both biological variability and technical factors [36,51]. Delayed sample processing, inappropriate storage conditions, reduced cell viability, and suboptimal stimulation protocols may all contribute to absent or diminished basophil activation, underscoring the importance of rigorous quality control throughout the analytical process [46,47,48].

In practical terms, distinguishing true biological non-responsiveness from methodological artefacts is essential. In patients with inconclusive BAT findings, careful evaluation of sample quality, timing of analysis, and performance of positive controls should be undertaken before interpreting negative results as evidence against clinically relevant allergy. When appropriate, repeat testing or reassessment under optimized laboratory conditions may be considered [46,47,48,51].

**Figure 5:**
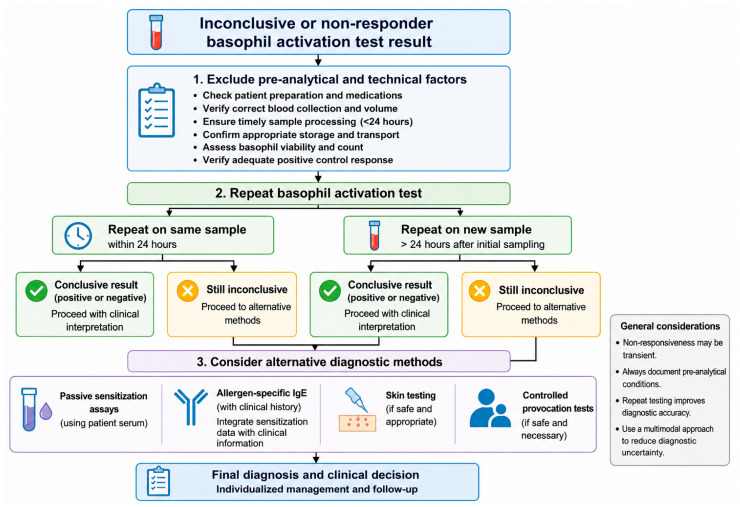
The proposed algorithm represents an evidence-informed conceptual approach rather than a validated clinical guideline. It includes evaluation of pre-analytical and technical factors, repeat testing under optimized conditions, and consideration of complementary diagnostic methods when clinically indicated. Final interpretation should be integrated with clinical history and conventional allergy testing. Created by the authors with AI-assisted graphical support using ChatGPT and manually revised based on the published evidence [36,46,47,48,51].

Importantly, non-responder status should not automatically be interpreted as evidence against clinically relevant hypersensitivity. Rather, inconclusive BAT findings should be interpreted cautiously and in conjunction with clinical history, conventional allergy testing, and alternative diagnostic strategies when necessary [36]. A simplified clinical approach to the interpretation of inconclusive or non-responder BAT results is presented in Figure 5.

## 7. Clinical Implications in Resource-Limited Settings and Precision Allergology

In resource-limited and emerging allergy care settings, including Kazakhstan and other Central Asian countries, BAT may provide additional diagnostic value in specialized centers managing complex cases of food allergy, drug hypersensitivity, polysensitization, and anaphylaxis. Although conventional diagnostic methods, including skin testing and allergen-specific IgE measurement, remain the cornerstone of allergy diagnosis, they do not always distinguish clinically relevant allergy from asymptomatic sensitization [3,53,54].

Current international recommendations recognize BAT as an emerging complementary functional assay in allergy diagnostics. EAACI has published dedicated guidelines, position papers, and consensus documents addressing BAT methodology, standardization, and clinical implementation [5,12,46,47,52]. In parallel, World Allergy Organization (WAO) guidance documents acknowledge BAT as an adjunctive laboratory investigation that may provide additional diagnostic information in selected diagnostically challenging clinical scenarios, while emphasizing that conventional diagnostic approaches remain the foundation of allergy diagnosis and patient management [55,56].

In Kazakhstan, national clinical protocols for anaphylaxis and drug hypersensitivity primarily rely on conventional allergy assessment but also recognize the role of additional in vitro immunological methods in selected diagnostically challenging situations. The national protocol for anaphylactic shock includes BAT, together with the cellular allergen stimulation test (CAST) and flow-cytometric cellular allergen stimulation test (FAST) modifications, as laboratory approaches that may be considered following severe allergic reactions when provocation testing is contraindicated or considered unsafe [57,58]. Local studies have also highlighted the potential utility of BAT-based approaches in selected cases of drug hypersensitivity and anesthetic-related allergic reactions, supporting their emerging role within tertiary allergy care [59,60]. 

Accordingly, BAT may be particularly valuable in patients with a history of severe allergic reactions, discordant results between clinical history and conventional testing, polysensitization, suspected immediate drug hypersensitivity, or when provocation testing is unsafe or impractical. In these situations, BAT may improve diagnostic confidence, reduce reliance on high-risk provocation procedures, and support more accurate identification of clinically relevant hypersensitivity [7,12,25,45,49].

Furthermore, because BAT requires specialized laboratory expertise, flow cytometry, standardized reagents, and strict quality control, it is best implemented as a targeted second-line diagnostic test rather than a routine screening tool [12,46,47,48,51]. A centralized testing model based on reference laboratories or specialized allergy centers may represent the most practical strategy for resource-limited healthcare systems by facilitating protocol harmonization, personnel training, quality assurance, and efficient use of laboratory resources [12,46,47].

Beyond analytical standardization, successful implementation of BAT also depends on healthcare system factors. Access to accredited reference laboratories, harmonized quality assurance procedures, and standardized testing protocols will be essential for broader clinical adoption across different healthcare settings [46,47,52]. In addition, future health-economic studies evaluating the cost-effectiveness of BAT and its potential to reduce unnecessary oral food or drug provocation tests may further support its integration into routine allergy practice.

Within the broader framework of precision allergology, BAT may contribute to more individualized diagnostic and therapeutic decision-making by providing functional evidence of clinically relevant sensitization and complementing conventional and molecular allergy diagnostics. However, broader implementation will depend on continued methodological standardization, improved accessibility, and validation in real-world clinical practice [3,6,8,12,61]. 

The current evidence supporting BAT across major allergic conditions varies according to the clinical indication, level of validation, and availability of standardized protocols. A comparative overview of the clinical utility of BAT in major allergic diseases is summarized in Table 3.

Although current evidence supports an expanding role for BAT in allergy diagnostics, several methodological and technological challenges remain before its broader implementation in routine clinical practice can be achieved.

## 8. Future Perspectives

Future developments in the basophil activation test are likely to depend on both technological innovation and methodological harmonization. The introduction of standardized commercial kits, automated analytical platforms, validated multiplex BAT formats, and artificial intelligence-assisted data analysis may improve accessibility, reproducibility, and clinical feasibility across different healthcare settings [46,47,61,62]. Practical considerations, implementation barriers, and future directions for BAT are summarized in Table 4.

Multiplex testing platforms capable of simultaneously assessing basophil reactivity to multiple allergens may improve diagnostic efficiency, particularly in polysensitized patients, while reducing sample volume requirements and laboratory workload. Combined with advances in automated flow cytometry, standardized gating strategies, and harmonized analytical protocols, these developments may enhance inter-laboratory reproducibility and facilitate broader clinical implementation of BAT [46,47,61,62].

Within the framework of precision allergology, BAT is expected to complement molecular allergy diagnostics, clinical phenotyping, and biomarker-based approaches by providing functional evidence of clinically relevant sensitization. Future developments may also include integration of BAT with multi-omics technologies and advanced computational approaches, enabling more comprehensive characterization of allergic phenotypes, identification of novel biomarkers, and further refinement of precision allergy diagnostics. This may improve risk stratification, support individualized diagnostic and therapeutic decision-making, and facilitate monitoring of selected therapeutic interventions [3,6,8,12,61,63,64].

In parallel, the mast cell activation test (MAT) represents another emerging functional diagnostic approach. Similar to BAT, MAT is based on flow cytometric assessment of activation markers following allergen stimulation but additionally enables evaluation of selected non-IgE-mediated pathways through MRGPRX2 expression on mast cells. Although MAT remains primarily investigational, early studies suggest potential applications in food allergy, drug hypersensitivity, and mechanistic studies of immediate hypersensitivity reactions. Further standardization and multicenter validation will be required before its incorporation into routine clinical practice [65,66,67,68,69,70].

Another emerging approach involves humanized rat basophilic leukemia (RBL) reporter cell assays. These engineered FcεRI-expressing cell lines, sensitized with patient serum, provide functional assessment of allergen-specific IgE and may offer advantages in assay standardization, reproducibility, and long-term cell availability. However, RBL-based assays remain investigational and require further clinical validation before routine implementation in allergy diagnostics [71,72,73].

Ultimately, successful integration of BAT into routine clinical practice will depend on continued international efforts toward methodological harmonization, regulatory acceptance, external quality assurance, and multicenter validation. Standardized stimulation protocols, clinically validated cut-off values, and prospective real-world studies, including cost-effectiveness analyses, will be essential to define the optimal role of BAT and related functional cellular assays within evidence-based precision allergy diagnostics [3,6,37,46,47,52,61,62].

Beyond synthesizing the available evidence, this review provides a clinically oriented perspective on the role of BAT by integrating methodological considerations, diagnostic performance, implementation challenges, and emerging applications into a unified conceptual framework. The proposed evidence-informed diagnostic algorithms are intended to facilitate interpretation of BAT results and should be regarded as conceptual decision-support tools rather than validated clinical guidelines.

## 9. Study Limitations

This review has several limitations that should be considered when interpreting its conclusions. First, because of its narrative design, study selection was not based on a formal systematic methodology and may therefore be influenced by heterogeneity in publication types, patient populations, allergens, stimulation protocols, basophil identification strategies, and outcome measures. Consequently, direct comparison of reported diagnostic performance across studies should be interpreted cautiously. Furthermore, no quantitative meta-analysis was performed because of the substantial methodological heterogeneity across the included studies with respect to study design, patient populations, allergen preparations, stimulation protocols, activation markers, and outcome measures. In addition, because narrative reviews are inherently susceptible to publication bias and selective reporting, findings should be interpreted in the context of the available evidence base.

Second, the available evidence on BAT remains methodologically heterogeneous. Variability in allergen preparations, stimulation conditions, activation markers, gating strategies, and positivity thresholds may influence reported sensitivity and specificity estimates and limit comparability between studies. Differences in study design and patient selection may also affect the generalizability of findings to routine clinical practice.

In addition, practical barriers also continue to restrict broader implementation of BAT. These include the requirement for fresh blood samples, specialized flow cytometry infrastructure, trained personnel, and careful pre-analytical handling. Furthermore, the presence of non-responder patients may reduce interpretability of results in a subset of individuals and necessitates cautious clinical interpretation. An additional logistical limitation is the requirement for rapid sample processing, as BAT generally relies on fresh blood specimens analyzed within a few hours of collection. This requirement may restrict access to testing in healthcare systems lacking specialized laboratory infrastructure or centralized allergy diagnostic facilities.

Despite these limitations, the available evidence supports the role of BAT as a clinically relevant complementary tool in selected cases of IgE-mediated allergy. However, further multicenter validation studies, methodological harmonization, and real-world implementation data will be necessary to better define its optimal clinical positioning.

## 10. Conclusions

The basophil activation test represents a clinically relevant functional assay that may provide additional diagnostic value in selected IgE-mediated allergic diseases. By assessing allergen-induced basophil responsiveness, BAT may help distinguish clinically relevant allergy from asymptomatic sensitization, particularly in diagnostically challenging cases.

Current evidence supports the use of BAT primarily as a complementary second-line diagnostic tool, especially in food allergy, drug hypersensitivity, Hymenoptera venom allergy, and patients at increased risk of severe allergic reactions. However, its broader implementation remains dependent on greater methodological standardization, validated interpretation criteria, and improved accessibility.

Within the evolving landscape of precision allergology, BAT may increasingly complement conventional and molecular diagnostic approaches to support more individualized clinical decision-making.

## Figures and Tables

**Table 1:** Characteristics of Representative Original Clinical Studies Included in the Narrative Review.

Author, Year	Country	Study Design	Population	Sample Size	BAT Markers	Key Findings	Clinical Implication
Santos et al., 2021 [13]	United Kingdom, Switzerland	Prospective multicenter diagnostic study (Pronuts Study)	Children (6 months–16 years) with confirmed peanut, tree nut, or sesame allergy undergoing evaluation for additional nut/seed allergies	90 children (83 analyzed; 7 non-responders excluded from ROC analysis)	CD63	BAT achieved 96–100% diagnostic accuracy and reduced the need for oral food challenges in children with nut and sesame allergy.	Supports BAT as a second-line diagnostic test in equivocal food allergy cases.
Duan et al., 2021 (MONAS) [14]	Canada, Austria	Prospective multicenter diagnostic accuracy study	Children aged 6 months–17 years with confirmed peanut and/or tree nut allergy or sensitization undergoing allergy evaluation and oral food challenges	200 enrolled; 159 with valid BAT results	CD63 (%CD63+ basophils on CCR3+ basophils)	BAT showed excellent diagnostic accuracy for peanut and tree nut allergy (AUROC 0.92–0.98), outperforming sIgE.	May reduce reliance on OFCs in multisensitized children.
Karadağ et al., 2025 [15]	Turkey	Prospective observational diagnostic study	Children with egg allergy undergoing OFC	46 children	CD63	BAT showed higher positivity in IgE-mediated egg allergy and greater specificity than SPT.	Useful adjunct before OFC in suspected IgE-mediated egg allergy.
Nguyen et al., 2025 [16]	Vietnam	Prospective diagnostic accuracy study	Adults with confirmed shrimp and/or prawn allergy	43 patients	CD63	BAT outperformed SPT and sIgE for shrimp allergy diagnosis (AUC up to 0.88).	May improve diagnostic certainty and reduce OFC requirements.
Céspedes et al., 2023 [17]	Spain	Prospective diagnostic study	Patients with suspected immediate hypersensitivity reactions to amoxicillin and/or amoxicillin–clavulanate	147 (105 amoxicillin allergy; 42 clavulanate allergy)	CD63, CD203c	CD203c demonstrated higher confirmatory value than CD63 for amoxicillin hypersensitivity.	Supports BAT as an adjunctive test in β-lactam allergy.
Reitmajer et al., 2024 [18]	Germany	Retrospective diagnostic study	Patients with suspected immediate β-lactam allergy	34 patients (37 BATs)	CD63	BAT showed high specificity (92.3%) but low sensitivity in β-lactam allergy.	Most useful as a confirmatory rather than screening test.
Krawiec et al., 2023 [19]	UK	Prospective diagnostic accuracy study (STARD-compliant)	Children (6 months–15 years) with suspected egg allergy undergoing DBPCFC	150	CD63	BAT showed the highest diagnostic performance among tested modalities for baked egg allergy and reduced the need for oral food challenges by approximately 41%; diagnostic performance was particularly strong in children < 2 years.	BAT may reduce the number of diagnostic food challenges while maintaining high diagnostic accuracy in pediatric egg allergy.
Bartha et al., 2025 [20]	UK	Prospective diagnostic accuracy study	Children with suspected cow’s milk allergy undergoing OFC	150	CD63, CD203c	BAT showed the highest diagnostic accuracy for baked and fresh cow’s milk allergy and markedly reduced OFC requirements.	Supports reduction of OFCs and improves diagnostic confidence in pediatric cow’s milk allergy.
Berin et al., 2018 [21]	USA	Multicenter prospective observational study	Children with challenge-confirmed egg allergy undergoing DBPCFC to baked egg and unheated egg	129 children (81 baked egg reactive, 48 baked egg tolerant)	CD63, CD203c	Higher egg-specific IgE and basophil activation were associated with baked egg reactivity, whereas T-cell responses did not differ between phenotypes.	BAT may help identify persistent egg allergy and predict baked egg tolerance.
Wai et al., 2021 [22]	Hong Kong, China	Prospective diagnostic accuracy study with DBPCFC reference standard	Patients aged 5–50 years with suspected shrimp allergy and documented reactions to shrimp	35 patients (15 DBPCFC-confirmed shrimp allergy; 20 shrimp-tolerant controls)	CD63, CCR3	BAT showed superior diagnostic accuracy for shrimp allergy (AUC 0.88, sensitivity 86.7%, specificity 94.1%) compared with SPT and shrimp-specific IgE.	BAT may replace multiple conventional tests and reduce the need for DBPCFC in patients with suspected shrimp allergy.
Cañas et al., 2022 [23]	Spain	Prospective diagnostic accuracy study	Adults with peach allergy sensitized to lipid transfer proteins (LTPs) and healthy controls	92 LTP-allergic patients + 16 healthy controls (total *n* = 108)	CD63, CD203c, CD-Sens, AUC	BAT accurately differentiated LTP-allergic patients from healthy controls, with sensitivity >87% and specificity >85%. BAT also identified peanut tolerance among LTP-sensitized individuals.	BAT may support the diagnosis of LTP-mediated food allergy and reduce the need for oral food challenges.
Deng et al., 2019 [24]	China	Prospective diagnostic accuracy study	Mugwort pollen-allergic patients with peach allergy, peach sensitization without symptoms, and non-allergic controls	69 participants (38 peach-allergic, 21 peach-sensitized tolerant, 10 controls)	CD63 (BAT to peach extract and Pru p 3)	BAT to Pru p 3 demonstrated high diagnostic accuracy (92.3% sensitivity, 94.6% specificity) and predicted reaction severity in mugwort pollen-related peach allergy.	BAT may support diagnosis and risk stratification in peach-allergic patients sensitized through mugwort pollen.
Czarnobilska et al., 2024 [25]	Poland	Prospective observational study	Adults with suspected drug-induced anaphylaxis (antibiotics, NSAIDs, local anesthetics, COVID-19 vaccines)	150 patients (90 underwent BAT)	CD63	BAT detected clinically relevant hypersensitivity to antibiotics, NSAIDs, local anesthetics, PEG, and COVID-19 vaccines, showing moderate-to-high diagnostic accuracy.	BAT may improve the safety of drug allergy work-up by helping identify culprit drugs and limiting the need for provocation testing.
García Ródenas et al., 2025 [26]	Spain	Prospective observational study	Adults with severe uncontrolled asthma initiating biologic therapy (omalizumab, mepolizumab, or benralizumab)	72 patients	CD63 (anti-IgE stimulated BAT)	Higher basophil reactivity was associated with poorer asthma control, lower treatment response rates, and more frequent biologic switching.	BAT may help predict response to biologic therapy and support treatment selection in severe asthma.
Boyd et al., 2025 [27]	United Kingdom	Prospective diagnostic cohort (BAT2 study)	Children undergoing baked milk and fresh milk oral food challenges	71 milk-allergic children	CD63, CD203c	BAT was the only biomarker able to distinguish severe from non-severe reactions and low- from high-threshold reactors. Sensitivity reached 71–96% with specificity up to 100% depending on reaction phenotype.	BAT may be used for risk stratification, helping identify patients at risk of severe reactions or reactions to very small allergen doses.

**Table 2:** Comparative Characteristics of BAT and Conventional Allergy Diagnostic Methods.

Diagnostic Method	Biological Target	Diagnostic Strengths	Major Limitations	Optimal Clinical Scenario	Diagnostic Role
SPT [4,5,44]	Immediate cutaneous sensitization	Rapid, inexpensive, widely available	Antihistamine interference; risk of systemic reactions; false-positive sensitization in polysensitized individuals	Initial allergy evaluation	First-line screening
Serum allergen-specific IgE [4,5,38]	Immunological sensitization	Standardized, reproducible, safe	Limited ability to distinguish sensitization from clinically relevant allergy	Baseline laboratory assessment	Complementary first-line test
BAT [3,7,8,12,45]	Functional basophil responsiveness	High specificity; reflects clinically relevant allergy; useful in equivocal cases	Requires fresh blood, flow cytometry, and technical expertise; non-responder phenomenon	Complex or inconclusive diagnostic scenarios	Second-line functional diagnostic tool
Provocation testing (OFC/DPT) [5,37,38,45]	Clinical reactivity	Reference diagnostic standard; confirms clinical allergy	Risk of severe reactions; resource-intensive; time-consuming	Persisting diagnostic uncertainty	Confirmatory reference test

Abbreviations: BAT—basophil activation test; SPT—skin prick testing; OFC—oral food challenge; DPT—drug provocation test.

**Table 3:** Comparative Clinical Utility of BAT Across Major Allergic Conditions.

Clinical Application	Evidence Strength	Reported Sensitivity	Reported Specificity	Main Advantage	Main Limitation	Potential Role
Food allergy [13,14,15,27]	High	80–95%	85–98%	High specificity, reduction of OFCs	Lack of standardization	Second-line diagnostic test
Drug hypersensitivity [17,18,45]	Moderate	Variable (20–80%)	80–100%	Useful in selected reactions	Variable sensitivity	Adjunctive test
Venom allergy [49,50]	High	85–100%	80–100%	Supports diagnosis in equivocal cases	Limited availability	Complementary tool
Latex allergy [3,8]	Moderate	Limited evidence	High specificity reported	Functional confirmation of sensitization	Limited evidence base	Specialized testing
AIT monitoring [11,26,43]	Emerging	Not established	Not established	Assessment of immunologic changes	Lack of validated thresholds	Research and selected clinical use

**Table 4:** Practical Considerations, Limitations, and Future Perspectives of the BAT.

Domain	Key Considerations	Clinical Implication
Pre-analytical factors [46,47,48]	Fresh blood samples should ideally be analyzed within 4 h of collection; delayed processing reduces basophil responsiveness and analytical reliability	Requires standardized logistics and rapid sample transport
Technical requirements [3,8,46,47]	Flow cytometry and trained personnel needed	Limited availability outside tertiary centers
Standardization [12,45,46,47]	Lack of universal cut-offs and protocols	Results may vary across laboratories
Biological variability [36,51]	Non-responder phenotype	Negative BAT does not fully exclude allergy
Clinical utility [7,12,13,37]	Particularly useful in polysensitized or equivocal cases	Helps reduce unnecessary provocation tests

## Data Availability

No new data were created or analyzed in this study.
